# Induced Production of 1-Methoxy-indol-3-ylmethyl Glucosinolate by Jasmonic Acid and Methyl Jasmonate in Sprouts and Leaves of Pak Choi (*Brassica rapa* ssp. *chinensis*)

**DOI:** 10.3390/ijms140714996

**Published:** 2013-07-18

**Authors:** Melanie Wiesner, Franziska S. Hanschen, Monika Schreiner, Hansruedi Glatt, Rita Zrenner

**Affiliations:** 1 Department of Quality Research, Leibniz-Institute of Vegetable and Ornamental Crops Grossbeeren and Erfurt e.V., Theodor-Echtermeyer-Weg 1, 14979 Grossbeeren, Germany; E-Mails: wiesner@igzev.de (M.W.); hanschen@igzev.de (F.S.H.); schreiner@igzev.de (M.S.); 2 Department of Nutritional Toxicology, German Institute of Human Nutrition Potsdam-Rehbrücke, Arthur-Scheunert-Allee 114-116, 14558 Nuthetal, Germany; E-Mail: glatt@dife.de

**Keywords:** *Brassica rapa* ssp. *chinensis*, signaling molecules, methyl jasmonate, 1-methoxy-indol-3-ylmethyl glucosinolate, glucosinolate biosynthesis pathway, indole glucosinolates, gene expression

## Abstract

Pak choi plants (*Brassica rapa* ssp. *chinensis*) were treated with different signaling molecules methyl jasmonate, jasmonic acid, linolenic acid, and methyl salicylate and were analyzed for specific changes in their glucosinolate profile. Glucosinolate levels were quantified using HPLC-DAD-UV, with focus on induction of indole glucosinolates and special emphasis on 1-methoxy-indol-3-ylmethyl glucosinolate. Furthermore, the effects of the different signaling molecules on indole glucosinolate accumulation were analyzed on the level of gene expression using semi-quantitative realtime RT-PCR of selected genes. The treatments with signaling molecules were performed on sprouts and mature leaves to determine ontogenetic differences in glucosinolate accumulation and related gene expression. The highest increase of indole glucosinolate levels, with considerable enhancement of the 1-methoxy-indol-3-ylmethyl glucosinolate content, was achieved with treatments of sprouts and mature leaves with methyl jasmonate and jasmonic acid. This increase was accompanied by increased expression of genes putatively involved in the indole glucosinolate biosynthetic pathway. The high levels of indole glucosinolates enabled the plant to preferentially produce the respective breakdown products after tissue damage. Thus, pak choi plants treated with methyl jasmonate or jasmonic acid, are a valuable tool to analyze the specific protection functions of 1-methoxy-indole-3-carbinole in the plants defense strategy in the future.

## 1. Introduction

**Figure 1:**
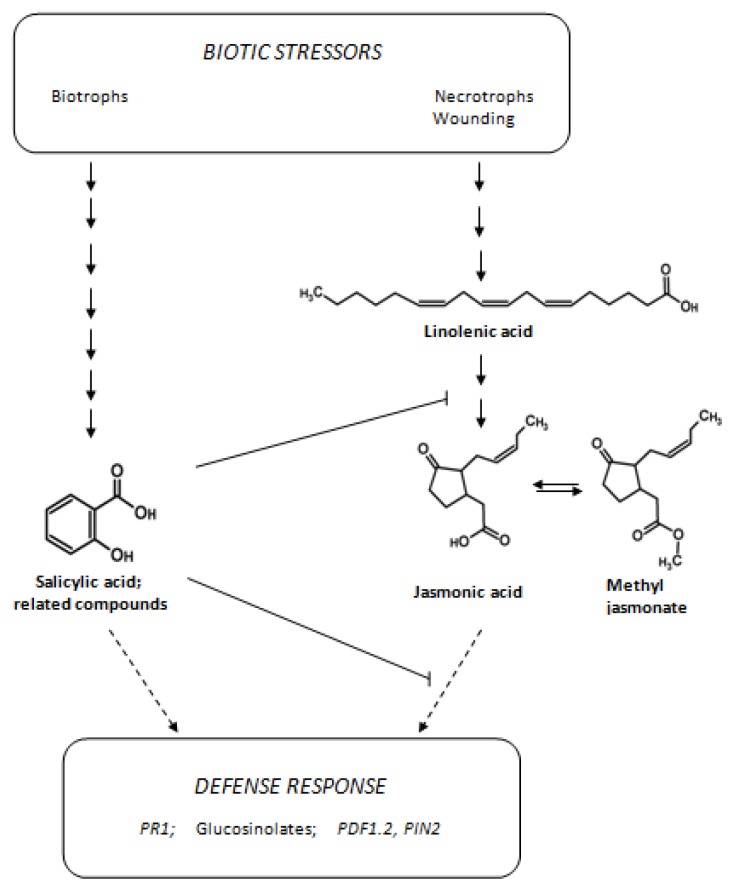
Signaling molecules involved in defense against biotic stressors. Investigated molecules are marked in bold. *PR1*, PATHOGENESIS-RELATED 1; *PDF1.2*, DEFENSIN-LIKE 16; *PIN2*, PROTEINASE INHIBITOR II.

Plants are sessile and therefore had to develop different strategies against multiple environmental impacts such as abiotic and biotic stressors. A very effective strategy against unwanted biotic effectors such as pathogens or herbivores is the accumulation of defense compounds [1]. The induction or increased synthesis of such compounds is mediated by signaling molecules, for instance jasmonic acid (JA), ethylene, and salicylic acid (SA), which activate corresponding transduction pathways. Necrotrophic pathogens and herbivorous insects are commonly deterred by jasmonate-dependent defenses, while pathogens with a biotrophic lifestyle are more sensitive to a salicylate-dependent response (Figure 1) [2–4].

Glucosinolates are characteristic secondary metabolites of the order Brassicales and can be regarded as defense compounds. Glucosinolates are β-d-thioglucoside-*N*-hydroxysulfates linked with a variable side chain, which is derived from different amino acid precursors. More than 120 glucosinolates are known [5], and depending on the chemical structure of the precursor amino acid they are classified into three groups: aliphatic, indole and aromatic glucosinolates. The glucosinolate profile itself and modifications thereof, together with specific hydrolyzation products, are being discussed as a defense mechanism of plants to cope with various abiotic and biotic stresses.

Since the discovery that a degradation product of 4-methylsulfinylbutyl glucosinolate is a strong inducer of phase II detoxification enzymes in mammals thus protecting against the risk of cancer [6,7], research in the last two decades has been mainly focused on the elucidation of aliphatic glucosinolates biosynthesis and their corresponding breakdown products [8–11]. Less attention has been paid to biosynthesis of indole glucosinolates, although these glucosinolates are induced by insect or pathogen attacks [12–15] and also influence the oviposition of crucifer specialist insects [16]. Indole glucosinolates were most prominent in roots and also found in leaves [17], therefore Brassicaceae providing breakdown products of glucosinolates are a good source for plant based manure and biofumigation, respectively [18]. Additionally, recent studies have shown that indole glucosinolates, especially breakdown products of 1-methoxy-indol-3-ylmethyl glucosinolate, exert mutagenic or genotoxic effects in mammalian and bacterial cell studies [19,20]. Thus, *Brassica* species with high indole glucosinolate concentrations could act specifically as a deterrent or toxin against insects or soilborn pathogens.

**Figure 2:**
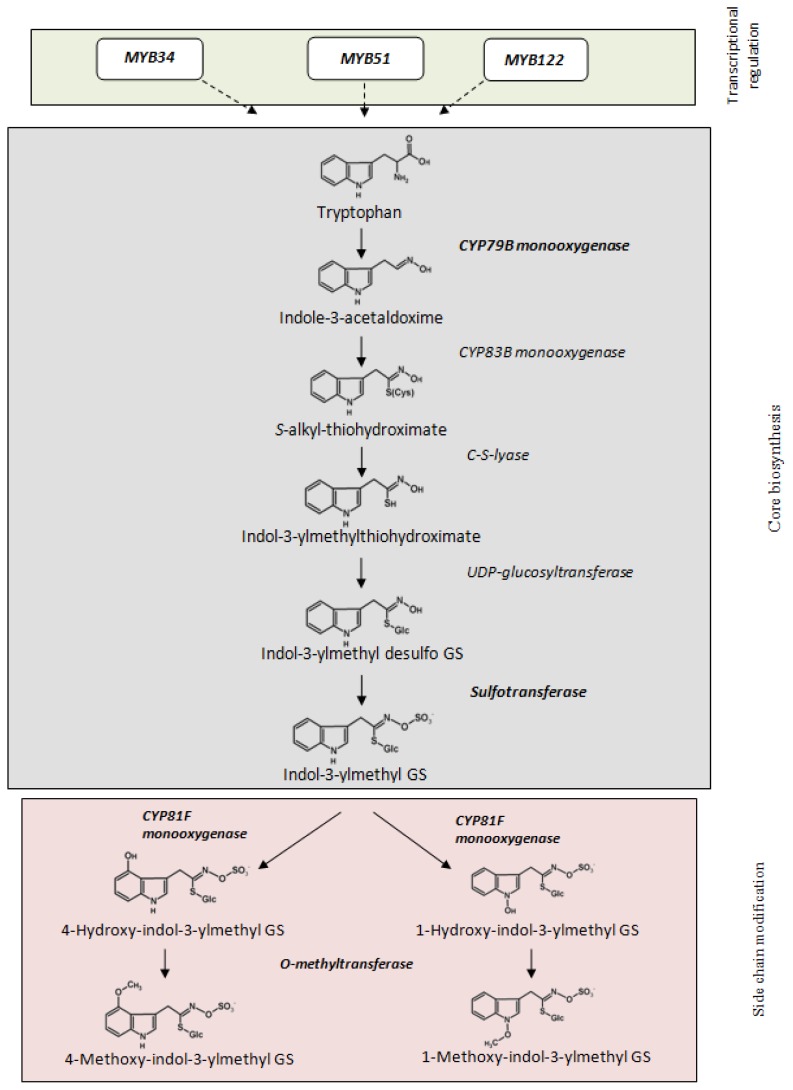
Biosynthesis of indole glucosinolates in Arabidopsis. Transcriptional regulators, enzymatic steps involved in core biosynthesis and side chain modification are shown. Steps analyzed in more detail on gene expression level are marked in bold.

Biosynthesis of indole glucosinolates starts with conversion of the precursor amino acid tryptophan to the corresponding aldoxime by cytochrome P450 monooxygenase CYP79B2 or CYP79B3 (Figure 2; [10]). The aldoxime enters the glucosinolate core biosynthesis to form the indol-3-methyl-desulfoglucosinolate and is finally sulfated by the sulfotransferase SOT16 to form the proper glucosinolate. Modification of the built indol-3-ylmethyl glucosinolate by members of the cytochrome P450 family CYP81F leads to formation of hydroxy-indol-3-ylmethyl glucosinolates that could be methylated to methoxy-indol-3-ylmethyl glucosinolates by a specific class of plant family 2-*O*-methyltransferases [21].

Most of the knowledge about effects of signaling molecules on glucosinolates biosynthesis is known from studies with the model plant *Arabidopsis thaliana* [22]. However, elicitation studies with signaling molecules, such as linolenic acid, methyl jasmonate, jasmonic acid and salicylic acid have also shown an enhancement of glucosinolates levels in several *Brassica* species [23–29]. It is known from *Arabidopsis thaliana* that methyl jasmonate induces the transcription factors *ATR1/MYB34* and *HIG1/MYB51* [30,31] subsequently altering the expression levels of *CYP79B2* and *CYP79B3* and the *SOT16* finally resulting in enhanced indol-3-ylmethyl glucosinolate levels [22]. In contrast it is known that salicylic acid or related compounds can inhibit the transcriptional activation induced by jasmonate [32,33]. Furthermore, application of salicylic acid to *A. thaliana* led to induction of mainly aliphatic glucosinolates, and specifically to the accumulation of 4-methoxy-indol-3-ylmethyl glucosinolate [22]. A current survey revealed that the constitutive level of 1-methoxy-indol-3-ylmethyl glucosinolate is reasonably high in the species pak choi (*Brassica rapa* ssp. *chinensis*) when compared to other Brassicales. Moreover this high level is specified by genotypic variation and ontogenetically modified in sprouts and leaves [34].

Since the *B. rapa* genome was recently identified [35] we chose pak choi as most revelatory *Brassica* model plant to analyze the signaling and metabolic pathway of indole glucosinolates, particularly of 1-methoxy-indol-3-ylmethyl glucosinolate.

To investigate the specific function of 1-methoxy-indol-3-ylmethyl glucosinolate within the plant-environment interaction in more detail, our aims were (1) to assess whether signaling molecules specifically affect 1-methoxy-indol-3-ylmethyl glucosinolate biosynthesis and if so (2) to evaluate ontogenetic differences in the induction of 1-methoxy-indol-3-ylmethyl glucosinolates (3) to determine the impact of these signaling molecules on the expression of key genes of the indole glucosinolate biosynthesis pathway and corresponding transcriptional regulators, and finally (4) to rate the expected profile of indole glucosinolate degradation products upon tissue rupture for assessing the respective breakdown products.

## 2. Results and Discussion

The influence of different signaling molecules on indole glucosinolate biosynthesis has been analyzed in the leafy *Brassica* vegetable pak choi in conjunction with gene expression studies of the core biosynthetic pathway (*CYP79B2/B3*, *SOT16*) and corresponding transcriptional regulators (*MYB34*, *MYB51*, *MYB122*), as well as genes putative involved in secondary modification of indol-3-ylmethyl glucosinolates (*CYP81F* family, *O*-methyltransferases).

Initially, a total of seven individual glucosinolates were determined in the pak choi cultivar Joi Choi. The group of aliphatic glucosinolate contained the alkenyl glucosinolates 3-butenyl, 4-pentenyl and one hydroxyalkenyl glucosinolate, 2-hydroxy-3-butenyl. The group of indole glucosinolates comprised the four indole glucosinolates indol-3-ylmethyl, 4-hydroxy-indol-3-ylmethyl, 4-methoxy-indol-3-ylmethyl, and 1-methoxy-indol-3-ylmethyl glucosinolate. While in pak choi sprouts all these mentioned glucosinolates were detectable, in mature leaves 2-hydroxy-3-butenyl and 4-hydroxy-indol-3-ylmethyl glucosinolate were below the detection limit.

### 2.1. Indole Glucosinolates Are Induced in Pak Choi Sprouts Treated with Signaling Molecules

**Figure 3:**
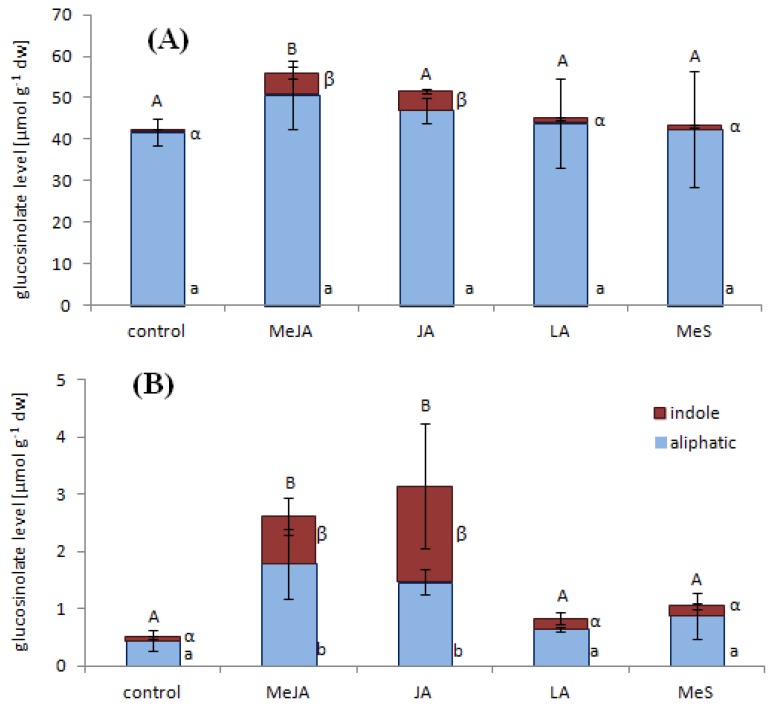
Glucosinolate levels in sprouts and mature leaves. Sprouts (**A**) and mature leaves (**B**) were harvested 48 h after elicitor or control treatment. Glucosinolate levels are shown as mean value with standard deviation (*n* = 5) of total aliphatic (blue) and total indole glucosinolates (red) in μmol g^−1^ dry weight (for details see Table S1). Elicitors: MeJA, methyl jasmonate; JA, jasmonic acid; LA, linolenic acid; MeS, methyl salicylate. Significant differences of total glucosinolate levels are labeled with different capital letters (**A**,**B**); significant differences of aliphatic glucosinolates are labeled with different Arabic letters (a,b); significant differences of indole glucosinolates are labeled with different Greek letters (α,β) (*p* ≤ 0.05, post-hoc Tukey’s HSD test).

The total glucosinolate level of untreated sprouts was 42.4 μmol g^−1^ dw with aliphatic glucosinolates describing 98% of the total glucosinolate level and only 2% were total indole glucosinolates. Depending on elicitor treatment the glucosinolate levels showed either no change or increased up to 56.1 μmol g^−1^ dw (Figure 3A), while plantlets were visibly not affected by any of the treatments. However, only methyl jasmonate and jasmonate treatment lead to a significant increase of indole glucosinolates (Figure 3A), whereas the aliphatic glucosinolates were less affected. The total indole glucosinolate level increased from 0.8 μmol g^−1^ dw prior to elicitor treatment to the highest amount of 5.2 μmol g^−1^ dw following methyl jasmonate treatment. Compared to control this equates up to an 8.5-fold enhancement of indole glucosinolates.

**Figure 4:**
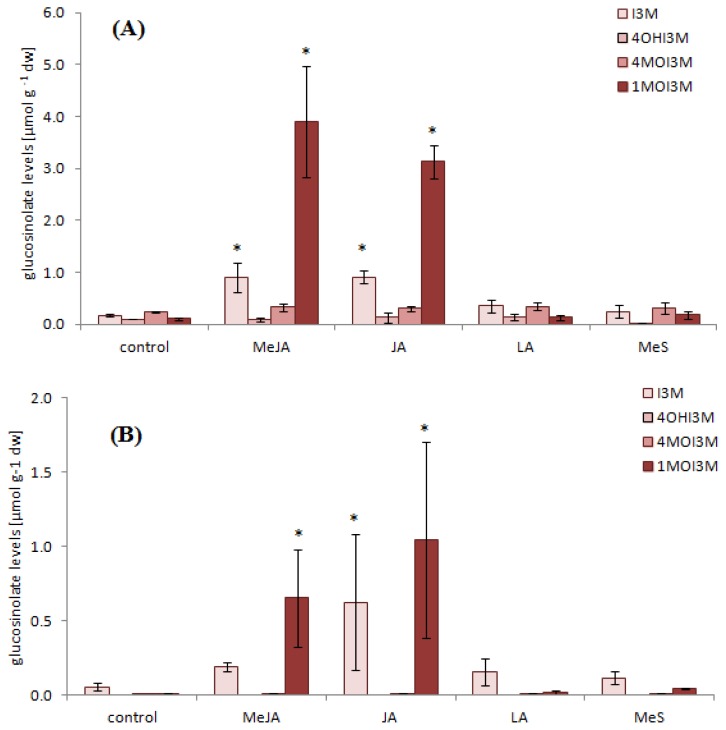
Individual indole glucosinolate levels in sprouts and mature leaves. Levels of indole glucosinolates shown as mean value with standard deviation (*n* = 5) in μmol g^−1^ dry weight of sprouts (**A**) and mature leaves (**B**) treated with signaling molecules: MeJA, methyl jasmonate; JA, jasmonic acid; LA, linolenic acid; MeS, methyl salicylate. *, treatment significantly different compared to control (*p* ≤ 0.05, Dunnett test). I3M, indol-3-ylmethyl glucosinolate; 4OHI3M, 4-hydroxy-indol-3-ylmethyl glucosinolate; 4MOI3M, 4-methoxy-indol-3-ylmethyl glucosinolate; 1MOI3M, 1-methoxy-indol-3-ylmethyl glucosinolate.

In addition, individual indole glucosinolates respond differently to the elicitor treatment in sprouts (Figure 4A). While 4-hydroxy-indol-3-ylmethyl and 4-methoxy-indol-3-ylmethyl glucosinolate remained almost unaffected with all different treatments, the application of specific elicitors had strong effects on indol-3-ylmethyl and 1-methoxy-indol-3-ylmethyl glucosinolate levels. Methyl jasmonate, jasmonate, and also linolenic acid induced indol-3-ylmethyl glucosinolate about 5-fold while linolenic acid led to a 2-fold increase, respectively. The strongest induction of 1-methoxy-indol-3-ylmethyl glucosinolate levels in sprouts of about 31-fold and 38-fold was detected after treatment with methyl jasmonate and jasmonate (Figure 4A).

These results are in agreement with other studies, reporting an enhancement of indole glucosinolates in *A. thaliana* and other Brassicaceae treated with signaling molecules [22–25,28,36]. Depending on the *Brassica* species the increase of total indole glucosinolate level after methyl jasmonate or jasmonic acid treatment was always based on an induction of indol-3-ylmethyl glucosinolate or its 1-methoxy modification product.

The induction of indole glucosinolate biosynthesis metabolism was further analyzed with expression studies of selected genes involved in this pathway and its regulation. As previously indicated more than one orthologue to genes of *A. thaliana* could be expected in the *Brassica rapa* genome [35]. Nucleotide sequences of coding regions of the genes of interest were identified on respective BAC clones of *Brassica rapa* cultivar Chiifu [37] and were used for the development of isoform specific oligonucleotide primer pairs (Table 1). The genes of interest were the transcriptional regulators of indole glucosinolate biosynthesis *MYB34*, *MYB51* and *MYB122*, the key genes of indole glucosinolate core biosynthesis, *CYP79B2/B3* and *SOT16*, and genes putatively involved in of side chain modification of indole-3-ylmethyl glucosinolate of the *CYP81F* gene family and selected members of the gene family of *O*-methyltransferases. Semi-quantitative real time RT-PCR was performed on RNA isolated from pak choi sprouts treated with methyl jasmonate, jasmonic acid, linolenic acid, and methyl salicylate, and were compared to respective control treatments. Specific amplification products were detectable with all chosen oligonucleotide primer combinations.

**Figure 5:**
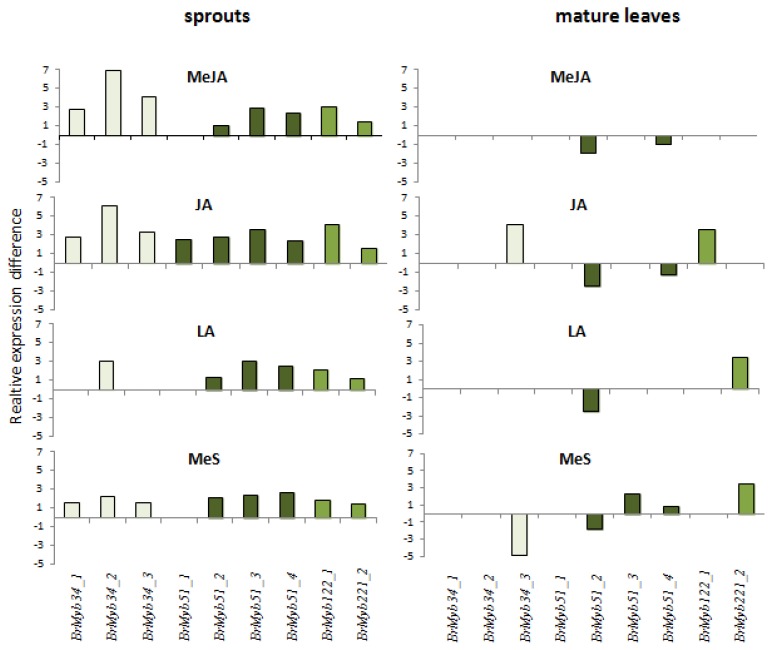
Relative expression difference of transcriptional regulators in sprouts and mature leaves. Left diagrams show expression in sprouts, right diagrams show expression in mature leaves treated with following elicitors: MeJA, methyl jasmonate; JA, jasmonic acid; LA, linolenic acid; MeS, methyl salicylate. Each value represents relative expression ratios calculated as ΔΔ*CT* from three biological replicates obtained from semi-quantitative RT-PCR analysis. For mapping the different abbreviations, please see Table 1. Only significant changes in gene expression levels after elicitor treatment compared to control plants are shown (Dunnett test, *p* < 0.05) (for details see basic data of gene expression levels in Table S2).

It is already known from experiments with *A. thaliana* that *AtMYB34* and *AtMYB51* are transcriptionally induced by methyl jasmonate [31]. Our results in pak choi sprouts are consistent with these findings. In detail, methyl jasmonate and jasmonate led to strong increases of *BrMYB34* depending on the isoform, and also the tested isoforms of *BrMYB51* are upregulated under these conditions (Figure 5). The treatments with linolenic acid and methyl salicylate also enhanced the expression of specific isoforms of *BrMYB34* and *BrMYB51* on pak choi sprouts, however, the increase was less pronounced. In addition, all elicitor treatments resulted in an induced expression of both isoforms of *BrMYB122* when compared to control sprouts (Figure 5).

**Figure 6:**
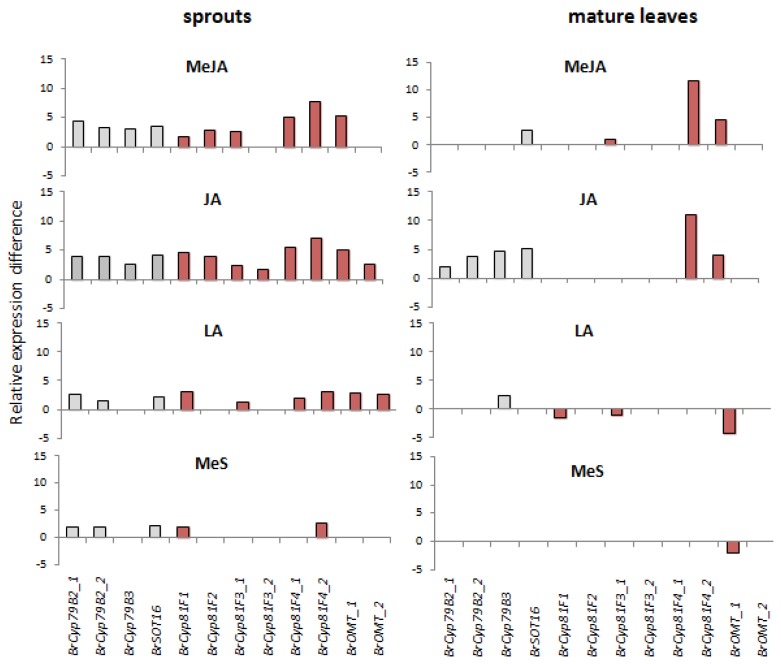
Relative expression difference of genes involved in indole glucosinolate biosynthesis in sprouts and mature leaves. Relative expression difference of genes involved in core biosynthesis of indole glucosinolates (light gray bars) and putative modifying genes (light red bars) are shown. Left diagrams show expression in sprouts, right diagrams show expression in mature leaves treated with following elicitors: MeJA, methyl jasmonate; JA, jasmonic acid; LA, linolenic acid; MeS, methyl salicylate. Each value represents relative expression difference calculated as ΔΔ*CT* from three biological replicates obtained from semi-quantitative RT-PCR analysis. For mapping the different abbreviations, please see Table 1. Only significant changes in gene expression levels after elicitor treatment compared to control plants are shown (Dunnett test, *p* ≤ 0.05) (for details see basic data of gene expression levels in Table S2).

In *A. thaliana* enhanced accumulation of indole glucosinolates is further accompanied by a jasmonate-dependent increase in expression of genes of the core biosynthetic pathway [22,38] as well as genes involved in tryptophan biosynthesis [39]. Similar effects of methyl jasmonate and jasmonate were found on transcriptional level for genes of the core biosynthetic pathway in pak choi sprouts (Figure 6). However, it is not possible to separate the direct effect of the chemical elicitor on the transcription of these genes from the influence caused by the various transcription factors since there are no respective mutants of pak choi available. Nevertheless, the expression of *BrCYP79B2/B3* isoforms and *BrSOT16* were strongly increased upon methyl jasmonate and jasmonate treatment, while linolenic acid and methyl salicylate led to only weak induction of *BrSOT16* and *BrCYP79B2* isoforms. The selected genes putatively involved in side chain modification of indol-3-ylmethyl glucosinolate showed also most expression differences with methyl jasmonate and jasmonate treatment when compared to the control.

**Figure 7:**
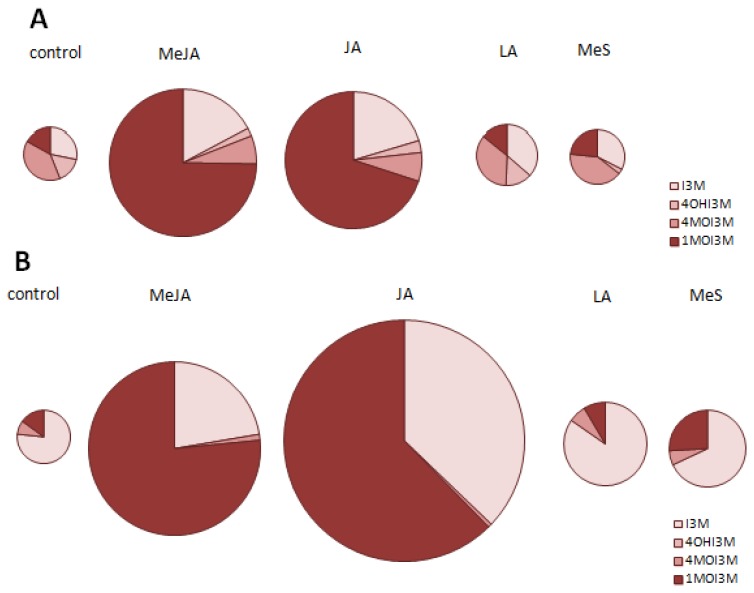
Individual glucosinolate levels as a percentage of the total indole glucosinolate level in sprouts and mature leaves. Percentage in sprouts (**A**) and leaves (**B**) are shown. The size of circle represents the amount of indole glucosinolates 48 h after the respective elicitor treatment. MeJA, methyl jasmonate; JA, jasmonic acid; LA, linolenic acid; MeS, methyl salicylate; I3M, indol-3-ylmethyl glucosinolate; 4OHI3M, 4-hydroxy-indol-3-ylmethyl glucosinolate; 4MOI3M, 4-methoxy-indol-3-ylmethyl glucosinolate; 1MOI3M, 1-methoxy-indol-3-ylmethyl glucosinolate.

Remarkably, in control sprouts the main indole glucosinolate is 4-methoxy-indol-3ylmethyl glucosinolate, followed by indol-3-ylmethyl and 1-methoxy-indol-3-ylmethyl glucosinolate (Figure 7A). As previously mentioned an increase of total indole glucosinolate levels was only found after treatment with methyl jasmonate or jasmonic acid and this was always based on an induction of indol-3-ylmethyl glucosinolate and its 1-methoxy modification product. Thus, with methyl jasmonate and jasmonate treatment the modification pattern of indol-3ylmethyl glucosinolate switched to 1-methoxy-indol-3-ylmethyl glucosinolate with a strong relative loss of 4-methoxy-indol-3-ylmethyl glucosinolate (Figure 7A). This finding supports the assumption that enzyme activity responsible for conversion of indol-3-ylmethyl glucosinolate to 4-methoxy-indol-3-ylmethyl glucosinolate or 1-methoxy-indol-3-ylmethyl glucosinolate might be specifically induced by signaling molecules [22]. Furthermore, experiments in *A. thaliana* have shown that methoxylation of indol-3-ylmethyl glucosinolate to 4-methoxy-indol-3-ylmethyl glucosinolate is suppressed by methyl jasmonate [22]. However, none of the selected genes putatively involved in side chain modification of indole glucosinolate is following such an expression pattern in pak choi sprouts.

### 2.2. Ontogenetic Differences in Induction of Indole Glucosinolates in Pak Choi

A previous survey of different pak choi cultivars revealed genotypic but also ontogenetic differences in the glucosinolate profile [34]. Therefore the same signaling molecules were also applied to mature leaves of pak choi plants and their effects on the glucosinolate levels together with the corresponding gene expression has been analyzed. The total glucosinolate level of untreated leaves was 0.5 μmol g^−1^ dw with aliphatic glucosinolates describing 86% of the total glucosinolate level and 14% were total indole glucosinolates. Depending on elicitor treatment the glucosinolate levels showed either no change or increased up to 3.1 μmol g^−1^ dw (Figure 3B), while leaves and whole plants were visibly not affected by any of the treatments in the timeframe investigated. Methyl jasmonate and jasmonate treatment lead to a significant increase of glucosinolates (Figure 3B), whereas in contrast to sprouts both the aliphatic and indole glucosinolates were affected. The total aliphatic glucosinolate levels increased from 0.4 μmol g^−1^ dw prior to elicitor treatment to the highest amount of 1.7 μmol g^−1^ dw following methyl jasmonate treatment, while the total indole glucosinolate level increased from 0.1 μmol g^−1^ dw to the highest amount of 1.7 μmol g^−1^ dw following jasmonic acid treatment. Compared to control this equates up to a 25-fold enhancement of indole glucosinolates. As in sprouts the strongest induction in leaves was detected in 1-methoxy-indol-3-ylmethyl glucosinolate levels after treatment with methyl jasmonate and jasmonate (Figure 4B). Although these results are in agreement with studies reporting an enhancement of indole glucosinolates in other Brassicaceae treated with signaling molecules the increases shown in our experiments are much higher than the data reported for example from *Brassica rapa* [40], *A. thaliana* [22] and *Brassica napus* [25].

The induction of indole glucosinolate biosynthesis metabolism in leaves was further analyzed with expression studies of the selected genes involved in this pathway in *A. thaliana* and its regulation. In contrast to our previous experiments with pak choi sprouts (Figure 5) the results in pak choi leaves did not show consistent and strong increases of *BrMYB34* and *BrMYB51* isoform expression 48 h after elicitor application (Figure 5). Only in the treatment with jasmonic acid an increased expression of *BrMYB34_3* and *BrMYB122_1* was found 48 h after application, while linolenic acid and methyl salicylate still enhanced the expression of *BrMYB122_2*. The absence of induced expression of the respective transcriptional regulators, the responsive elements that initiate transcriptional answers on external stimuli, could be due to the sampling of plant material with 48 hours adaptation time following elicitor treatment. While this timeframe is necessary to see maximum changes in the glucosinolate profile of sprouts [41], it might be too late to analyze induction of transcription factors involved [31,42]. Harvesting of leaves at earlier time points after elicitor application would be needed for confirmation; however it is not the primary focus of this study. Additionally, the transcriptional level of genes of the core biosynthetic pathway was analyzed and induced expression of *BrCYP79B2/B3* isoforms and *BrSOT16* was still detectable 48 hours after jasmonate application (Figure 6). This is in good agreement with the finding that jasmonate treatment causes the strongest induction of indole glucosinolates (Figure 4B). More specific as in sprouts only one of the selected genes putatively involved in side chain modification of the *CYP81F* gene family and of *O*-methyltransferases showed significant expression differences with methyl jasmonate and jasmonate treatment when compared to the control.

When compared to sprouts treatment with methyl jasmonate or jasmonic acid strongly induces indole glucosinolate biosynthesis also in pak choi leaves. This induction is accompanied by an extraordinary enhancement of its 1-methoxy indol-3-ylmethyl glucosinolate (Figure 7B). Although there are numerous reports showing an enhancement of indole glucosinolates in Brassicaceae treated with signaling molecules [22–28] the increase of 1-methoxy indol-3-ylmethyl glucosinolate shown in our experiments are much higher.

### 2.3. High Levels of Particular Indole Glucosinolate Breakdown Products after Tissue Disruption of Pak Choi Leaves

It is reported that hydrolysis products of indole glucosinolates are bioactive compounds that might act as deterrent or toxin against insects or pathogens [43]. Therefore plants with high indole glucosinolate content could be useful to control pests in a process known as biofumigation [18]. In this regard, pak choi plants with greatly enhanced indole glucosinolate levels were analyzed for their ability to produce particular hydrolysis compounds of indole glucosinolates. The content and composition of these degradation products was determined by GC-MS. Besides indole-3-acetonitrile also degradation products of 1-methoxy-indol-3-ylmethyl glucosinolate, namely 1-methoxy-indole-3-acetonitrile as well as 1-methoxy-indole-3-carbinol were detected from homogenized pak choi leaves (Table 2). Due to its reactivity it was anticipated not to find any isothiocyanate breakdown products of indole glucosinolates [44], as they are very unstable and immediately form the respective carbinole and thiocyanate. As expected, the levels of indole glucosinolate breakdown products were significantly higher in pak choi plants treated with methyl jasmonate when compared to controls and sum up to 66% of all glucosinolate degradation products determined. Although more 1-methoxy-indol-3-ylmethyl glucosinolate than indol-3-ylmethyl glucosinolate occurred in treated plants, breakdown products from 1-methoxy-indol-3-ylmethyl glucosinolate were lower in concentration (Table 2). This could be attributed to further breakdown and reactions of the formed 1-methoxy-indole-3-carbinole, as indole-3-carbinoles are reported to be very reactive compounds [43]. Moreover, contrasting to the control methyl jasmonate, treated plants generated an additional breakdown product - the 1-methoxy-indole-3-carbinole (Table 2). According to Glatt *et al.* [20] the alcohol of 1-methoxy-indol-3-ylmethyl glucosinolate is the most mutagenic breakdown product suggesting that 1-methoxy-indole-3-carbinole might be a prominent compound in the plants defense strategy.

Although indole glucosinolates are very common in Brassicaceae, glucosinolates with aliphatic side chains are usually dominating the chemical profile of cruciferous plants. This might be the reason that up to date indole glucosinolates are less explored for their impact on plant-insect interaction [43]. However, recent studies using transgenic *A. thaliana* plants with increased indole glucosinolates and a doubling of 1-methoxy-indole-3-ylmethyl glucosinolate [45] indicated specific differences in oviposition and survival rate of the phloem feeding insect *Bemisia tabaci* [46]. There is also increasing evidence that the function of indole glucosinolates can be extended to defense against non-host fungal pathogens [47,48]. In future experiments pak choi plants with highly increased indole glucosinolate levels will be a valuable tool to analyze the specific defense function of 1-methoxy-indol-3-ylmethyl glucosinolate and its breakdown products in a natural setting with a variety of herbivores and pathogens. In addition, the specific contribution of 1-methoxy-indol-3-ylmethyl glucosinolate to biofumigation could be tested in different agricultural cultivation systems.

## 3. Experimental Section

### 3.1. Plant Material

Seeds of *Brassica rapa* ssp. *chinensis* (pak choi) cultivar Joi Choi (Enza Zaden, Germany) were sown on bars of fleece, containing 3 g seeds per fleece, placed in aluminum foil trays (33 × 10 cm) filled with perlite. These were kept in a green house chamber with 12 h photoperiod (220 μmol m^−2^ s^−1^ of photosynthetic active radiation) and a temperature regime of 24/20 °C (day/night) at relative humidity about 75%. The sprouts were watered as needed, no fertilizer was added.

Additionally, seeds were sown in a sowing pan. After 10 days seedlings were transplanted to 10 L pots and grown under environmentally controlled greenhouse conditions (20 °C, 300 μmol m^−2^ s^−1^ photosynthetic active radiation, and relative humidity 60%–75%).

### 3.2. Elicitors and Plant Treatment

Methyl jasmonate, jasmonic acid, linolenic acid, and methyl salicylate were selected as elicitors. The different concentrations were selected according to previous studies. All elicitors (Sigma Aldrich, Taufkirchen, Germany) were resolved in water containing 0.01% (*v*/*v*) Tween20 to reduce surface tension and were applied as follows: methyl jasmonate (MeJA, 200 μM), jasmonic acid (JA, 200 μM), methyl salicylate (MeS, 200 μM), and linolenic acid (LA, 200 μM). Water containing 0.01% (*v*/*v*) Tween20 was sprayed as control treatment.

The 10 days old sprouts were treated with chemical elicitors by spraying each bar of fleece with 15 mL of the respective elicitor solution. 48 h after treatment the total aerial tissue was harvested. Seedlings were cut at the substrate level, and the fresh weight of tissue was recorded. Samples were frozen at −50 °C, subsequently lyophilized, blended to a fine powder, and stored until analysis. For each treatment, five samples were taken as replicates.

Fully developed plants with 9 to 11 leaves were treated with elicitors in same concentrations as mentioned before. Each plant was sprayed with approximately 15 mL of the respective elicitor or control solution. 48 h after treatment the plants were harvested by cutting the leaves and removing the midrib. Samples were frozen at −50 °C, subsequently lyophilized, blended to a fine powder, and stored until analysis. For each treatment five samples were taken as replicates.

### 3.3. Sample Preparation and Desulfo-Glucosinolate Analysis by HPLC

Glucosinolate concentration was determined as desulfo-glucosinolates using a modified method according to DIN EN ISO 9167-1, described previously in Wiesner *et al.* [34]. Twenty mg of powdered samples were extracted and analyzed by HPLC-DAD using a Merck HPLC system (Merck-Hitachi, Darmstadt, Germany) with a Spherisorb ODS2 column (Bischoff, Leonberg Germany; particle size 5 μm, 250 mm × 4 mm). Desulfo-glucosinolates were identified based on comparison of retention times and UV absorption spectra with those of known standards. Glucosinolate concentration was calculated by the peak area relative to the area of the internal standard. Results are given as micromoles per gram dry weight. Glucosinolate concentration was determined in 5 replicates, each replicate sample measured in duplicate.

### 3.4. Gene Expression Analysis by Semi-Quantitative Realtime RT-PCR

RNA was extracted from 100 mg tissue using the NucleoSpin Plant Kit (Macherey-Nagel GmbH and Co KG, Düren, Germany), including on-column DNaseI digestion. RNA was quantified spectrophotometrically at 260 nm (Nanodrop ND1000, Technology Inc., Wilmington, DE, USA), and quality was checked using the ratio of absorption at 260 and 280 nm with a ratio between 1.9 and 2.1 as acceptable. Single-stranded cDNA synthesis was carried out with total RNA using SuperScript™ III RNaseH–reverse transcriptase (Invitrogen GmbH, Karlsruhe, Germany) with oligo d(T12–18) primers according to the manufacturer’s instructions. Gene-specific primer sets are listed in Table 1. PCR amplified sequences generated with these oligonucleotide primer pairs and cDNA from pak choi leaves as template were subcloned and verified by sequence analysis. Semi-quantitative two-step RT-PCR was performed using a SYBR^®^ Green 1 protocol in 96-well reaction plates on an Applied Biosystems 7500 Realtime PCR System. The following thermal profile was used for all reactions: 50 °C for 2 min, 95 °C for 10 min, 40 cycles of 95 °C for 30 s and 60 °C for 1 min, followed by dsDNA melting curve analysis to ensure amplicon specificity. Each reaction was done in a 10 μL volume containing 200 nM of each primer, 3 μL of cDNA (1:50) and 7 μL of Power SYBR Green Master Mix (Applied Biosystems, Grand Island, NY, USA). Data generated by semi-quantitative real-time PCR were collected and compiled using 7500 v2.0.1 software (Applied Biosystems, Grand Island, NY, USA). Data were exported to LinReg software [49] to determine the PCR amplification efficiency for each primer pair. Relative transcript levels were normalized on the basis of expression of an invariant control orthologous to At3g18780, *ACT2*, on KBrB071H12 using the equation Δ*CT* [50], where Δ*Ct* is the difference between control and target products (Δ*Ct* = *Ct*_gene_ − *Ct*_act_). Semi-quantitative PCR was performed on at least three biological replicates measured in duplicates for each gene, and non-template controls were included. Thus, the calculated relative expression values are normalized to the control expression level (ΔΔ*Ct* = Δ*Ct*_treatment_ − Δ*Ct*_control_).

### 3.5. Analysis of Breakdown Products of 1-Methoxy-indol-3-ylmethyl Glucosinolate

For the determination of enzymatically formed breakdown products of indole glucosinolates, the method of Hanschen [51] was adapted. One milliliter of water was added to 500 mg of fresh plant tissue, ground in a centrifuge tube with a ball mill for 2 min at a frequency of 30 Hz (MM400 Retsch GmbH, Haan, Germany) and left for 30 min at room temperature for glucosinolate hydrolysis. 2 mL of methylene chloride (Carl Roth GmbH, Karlsruhe, Germany; GC Ultra Grade) and 100 μL of 2 mM benzonitrile in methylene chloride as internal standard (Sigma-Aldrich Chemie GmbH, Steinheim, Germany; ≥99.9%) were added and tubes were sealed. After shaking for 20 s and centrifugation for 5 min, the methylene chloride layer was removed and filtered through a small column of anhydrous sodium sulfate (VWR International GmbH, Darmstadt, Germany; ≥99%) to remove residual water. The remaining aqueous layer was re-extracted with 2 mL of methylene chloride. The dried extracts were combined, concentrated under nitrogen gas flow to 300 μL and transferred into a vial. Samples were analyzed by gas chromatography-mass spectrometry detection (GC-MS) using an Agilent 6890 A Series GC System (Agilent Technologies, Böblingen, Germany) with a Gerstel Multi Purpose Sampler MPS2 (Gerstel GmbH & Co. KG, Mühlheim, Germany) and an Agilent 5973 Network MSD. The GC was equipped with an Optima 5 MS column (Macherey-Nagel, Germany, 30 m × 0.25 mm × 0.25 μm film). After splitless injection of 1 μL of the sample at 190 °C, analytes were separated, using helium as carrier gas (1.8 mL min^−1^), and a temperature gradient starting at 35 °C (3 min) and raising up to 50 °C with 9 °C/min. After holding this temperature for 7 min, the temperature increased to 210 °C with 9 °C per min, then with 3 °C per min to 223 °C and finally with 35 °C per min to 310 °C. The temperature of the transfer line was 310 °C, the ion source of the MSD was set to 230 °C. Mass spectra were acquired in the EI+ (70 eV) full scan mode (TIC) (*m*/*z* 30–350). Analytes were identified by comparing mass spectra and retention times with those of authentic standards. Analyte content was calculated using TIC mode, benzonitrile as internal standard, and the response factor (*RF*) of each compound relative to benzonitrile. 1-methoxy-indole-3-carbinole and 1-methoxy-indole-acetonitrile standards were synthesized as reported previously [20]. The RFs were experimentally determined for indole-acetonitrile (Acros Organics: Thermo Fisher Scientific, Geel, Belgium; ≥98%) (*RF* = 0.84) and 1-methoxy-indole-3-carbinole (*RF* = 2.27). 1-methoxy-indole-acetonitrole was quantified with the *RF* of indole-acetonitrile, as the amount of provided standard did not suffice for *RF* determination. The limit of detection ranged between 1 μM (indole-acetonitrile) and 17.5 μM (1-methoxy-indole-3-carbinole).

### 3.6. Statistical Analysis

Statistical analyses were conducted with the software STATISTICA [52] (version 10, Statsoft Inc. Tulsa, OK, USA). The total glucosinolate level and the level of each desulfo-glucosinolate were subjected to analysis of variance (ANOVA) and post-hoc Tukey’s HSD test (*p* ≤ 0.05).

The significance in expression was compared to control using the analysis of variance (ANOVA) and post-hoc Dunnett’s test (*p* ≤ 0.05).

## 4. Conclusions

Treatment of pak choi sprouts and leaves with the signaling molecules methyl jasmonate and jasmonic acid resulted in strong increase of indole glucosinolates with major effect on 1-methoxy-indol-3-ylmethyl glucosinolate. On gene expression level this was accompanied by an upregulation of key genes involved in indole glucosinolate biosynthesis when compared to controls. Based on these results, it is assumed that genes specifically involved in side chain modification of indol-3-ylmethyl glucosinolate to synthesize 1-methoxy-indole-3-ylmethyl glucosinolate are highly induced by these signaling molecules.

The eliciting effects of signaling molecules on specific biosynthetic pathways might be used as a tool to develop *Brassica* species with desired glucosinolate profiles. Here a vegetable model plant has been described with strong enhancement of a single indole glucosinolate. Analysis of breakdown products after tissue damage has shown that in methyl jasmonate treated plants, 1-methoxy-indole-3-carbinole and the -acetonitrile were formed in high concentrations. In the future this model plant system could be used to analyze the causal relationship between enriched individual glucosinolate breakdown products and insect performance in studies testing toxic or repellent effects on biotic stressors, such as soilborne pathogens, aphids, trips, Lepidoptera and other herbivores.

## Supplementary Information



## Figures and Tables

**Table 1:** Oligonucleotide primers used for gene expression analysis by semi-quantitative realtime RT-PCR.

Gene function (*Gene name*)	Oligonucleotide abbreviation	Sequence	Accession (*Gene abbr.*)
Actin2 (*ACT2*)	Br-Af	ACGTGGACATCAGGAAGGAC	AC189447
	Br-Br	CTTGGTGCAAGTGCTGTGAT	(*BrACT2*)

Transcription factor Altered Tryptophan Regulation 1 (*ATR1*/*MYB34*)	Br-Myb34f1	GAAGGAATAAAGAAAGGAGCC	FJ584293
	Br-Myb34r1	GCATCTCTTCAATCCAGCTTTT	(*BrMYB34_1*)
	Br-Myb34f2	AGAAGGAATAAAGAAAGGAGCT	FJ584294
	Br-Myb34r1		(*BrMYB34_2*)
	Br-Myb34f2		FJ584295
	Br-Myb34r2	CATCTCTTCAATCCAGCTTTC	(*BrMYB34_3*)

Transcription factor High Indolic Glucosinolate 1 (*HIG1/MYB51*)	Br-Myb51f1	ACCGATAACGAAATCAAGAACTA	FJ584296
	Br-Myb51r1	CTCACAAGAACATATCAGAAAATT	(*BrMYB51_1*)
	Br-Myb51f2	ACCGATAACGAAATCAAGAACCA	FJ584297
	Br-Myb51r2	TCCGACAAATCAGAAAACCTCC	(*BrMYB51_2*)
	Br-Myb51f3		FJ584299
	Br-Myb51r2	ACCGATAACGAAATCAAGAACCT	(*BrMYB51_3*)
	Br-Myb51f1		FJ584298
	Br-Myb51r3	TCCGAAACCAGCAAATCAGAAA	(*BrMYB51_4*)

Transcription factor High Indolic Glucosinolate 2 (*HIG2/MYB122*)	Br-Myb122f1	TGGATGAATCTTCTTCAGACAAT	FJ584300
	Br-Myb122r1	ATGTCGCTTAATGATAGCCACC	(*BrMYB122_1*)
	Br-Myb122f2	TGGATGAATCTTGTTTGGAGAAA	Bra008131
	Br-Myb122r2	ACTATGTGTAGTGATAGTCGTG	(*BrMYB122_2*)

Cytochrom P450 monooxygenase family 79B (*CYP79B*)	Br-Cyp79B2f3	GTTCTCTGAAAACACTGCAGCG	FJ376045
	Br-Cyp79B2r3	TGCTTTTTGTATATCTGATTATCTAC	(*BrCYP79B2_1*)
	Br-Cyp79B2f4	GTTCTCTGAAAACACCGCACCT	FJ376047
	Br-Cyp79B2r4	CTCCTTTTGTATCTCTGATTATCTA	(*BrCYP79B2_2*)
	Br-Cyp79B3f	TTCTCGGAGAAAACCAAAACC	FJ376047
	Br-Cyp79B3r	TGCGTTTTGTGTATCGGACTA	(*BrCYP79B3*)

Sulfotransferase 16 (*SOT16*)	Br-SOT16f2	TTCAAGACGGCAAGAACCAG	FJ376059
	Br-SOT16r2	GGGTCAGCAGCTAGCGAG	(*BrSOT16*)

Cytochrom P450 monooxygenase family 81F (*CYP81F*)	Br-Cyp81F1f	TCCCTCGCACGCCGACG	KBrB006J12.9
	Br-Cyp81F1r	AGGATGCGGCAGCGAGTTA	(*BrCYP81F1*)
	Br-Cyp81F2f	TCTCCTTCTGAAGATCTCAAAA	KBrB027E01.6
	Br-Cyp81F2r	AGAAAAAGAAGCAGCGAACAC	(*BrCYP81F2*)
	Br-Cyp81F3f1	GCCGAGATCACCGATGGAA	KBrB006J12.6
	Br-Cyp81F3r1	GCGGAGGAGAAGACGTTCA	(*BrCYP81F3_1*)
	Br-Cyp81F3f2	GCCAAGATCGACGACAGAC	KBrH064I20.2
	Br-Cyp81F3r2	TCGGAGAAGGAGGAGAAGAC	(*BrCYP81F3_2*)
	Br-Cyp81F4f1	TTAACGGAAGAGGACATCAAAG	KBrB006J12.7
	Br-Cyp81F4r1	AAAGAGGGGAAGGAGACAAAGA	(*BrCYP81F4_1*)
	Br-Cyp81F4f2	TTAACAGTAGAGGACATCAAGA	KBrH064I20.1
	Br-Cyp81F4r2	TGGAGGAGAAGGAGAAAAGGA	(*BrCYP81F4_2*)

*O*-methyltransferase family protein (*OMT*)	Br-OMTf1	GGCTGTACCGGAGAGACGA	Bra017700
	Br-OMTr1	GCCGTTCTCATCAAGTGGGTG	(*BrOMT1_1*)
	Br-OMTf2	GGCTGTACCGGAGAGACCC	Bra017699
	Br-OMTr2	AACGTTTTCATCAAGTGGGTCT	(*BrOMT1_2*)

**Table 2:** Indole glucosinolate levels and corresponding breakdown products.

	Control	MeJA treated plants
Glucosinolate level (μmol g^−1^ dry weight)		

Aliphatic glucosinolates	2.80 ±1.24	6.20 ± 2.15
Indol-3-ylmethyl	0.26 ± 0.07	4.24 ± 0.73 *
4-Hydroxy-indol-3-ylmethyl	0.06 ± 0.02	0.21 ± 0.05 *
4-Methoxy-indol-3-ylmethyl	0.08 ± 0.01	0.11 ± 0.03
1-Methoxy-indol-3-ylmethyl	0.07 ± 0.04	5.84 ± 0.66 *

Breakdown products (μmol g^−1^ fresh weight)		

Aliphatic isothiocyanates, nitriles	0.251 ± 0.202	0.199 ± 0.120
Indole-acetonitrile	0.017 ± 0.018	0.246 ± 0.094 *
1-Methoxy-indole-acetonitrile	0.001 ± 0.003	0.079 ± 0.031 *
1-Methoxy-indole-3-carbinole	0.000 ± 0.000	0.057 ± 0.024 *

I3M, indol-3-ylmethyl glucosinolate; 4OHI3M, 4-hydroxy-indol-3-ylmethyl glucosinolate; 4MOI3M, 4-methoxy-indol-3-ylmethyl glucosinolate; 1MOI3M, 1-methoxy-indol-3-ylmethyl glucosinolate. MeJA, methyl jasmonate;

* , significantly different (*p* ≤ 0.05, post-hoc Tukey’s HSD test).
