# Supplementary material for: Induced Production of 1-Methoxy-indol-3-ylmethyl Glucosinolate by Jasmonic Acid and Methyl Jasmonate in Sprouts and Leaves of Pak Choi (Brassica rapa ssp. chinensis)
Source: Int J Mol Sci. 2013 Jul 18;14(7):14996–5016. doi: 10.3390/ijms140714996 (PMC3742284; doi:10.3390/ijms140714996)
Supplement: Supplementary file 1 [file ijms-14-14996-s001.pdf]

Supplementary Information

Table S1. Levels (μmol g^{-1} dry weight) of total aliphatic and indole glucosinolates, total glucosinolates, and individual indole glucosinolates in pak choi sprouts and mature leaves 48 h after application of the respective elicitors.

	**Total aliphatic**	**Total indole**	**Total GS**	**I3M**	**4OHI3M**	**4MOI3M**	**1MOI3M**
Sprouts							
Control	41.82 ± 3.17	0.61 ± 0.07	42.43 ± 3.18	0.17 ± 0.03	0.10 ± 0.01	0.24 ± 0.02	0.10 ± 0.03
MeJA	50.84 ± 8.22	5.22 ± 1.41	56.06 ± 9.60	0.90 ± 0.29	0.10 ± 0.03	0.32 ± 0.07	3.90 ± 1.07
JA	47.02 ± 3.11	4.47 ± 0.49	51.49 ± 3.55	0.91 ± 0.13	0.13 ± 0.11	0.30 ± 0.06	3.13 ± 0.31
LA	44.06 ± 10.74	0.95 ± 0.30	45.02 ± 11.03	0.35 ± 0.11	0.14 ± 0.06	0.34 ± 0.07	0.14 ± 0.05
MeS	42.47 ± 14.10	0.75 ± 0.30	43.22 ± 14.38	0.25 ± 0.11	0.02 ± 0.01	0.31 ± 0.11	0.18 ± 0.07
Mature leaves							
Control	0.439 ± 0.184	0.071 ± 0.03	0.510 ± 0.210	0.05 ± 0.03		0.006 ± 0.002	0.01 ± 0.00
MeJA	1.788 ± 0.604	0.832 ± 0.32	2.619 ± 0.891	0.17 ± 0.06		0.010 ± 0.003	0.65 ± 0.33
JA	1.469 ± 0.216	1.678 ± 1.19	3.147 ± 1.263	0.62 ± 0.46		0.009 ± 0.004	1.05 ± 0.66
LA	0.645 ± 0.028	0.185 ± 0.10	0.830 ± 0.102	0.16 ± 0.09		0.013 ± 0.005	0.02 ± 0.01
MeS	0.882 ± 0.407	0.168 ± 0.05	1.051 ± 0.440	0.11 ± 0.04		0.010 ± 0.004	0.04 ± 0.01

GS, glucosinolates; I3M, Indol-3-ylmethyl; 4OHI3M, 4-Hydroxy-indol-3-ylmethyl; 4MOI3M, 4-Methoxy-indol-3-ylmethyl; 1MOI3M, 1-Methoxy-indol-3-ylmethyl; MeJA, methyl jasmonate; JA, jasmonic acid; LA, linolenic acid; MeS, methyl salicylate. Each value represents the mean ± SD of five replicates.

Table S2. Expression levels of semi-quantitative realtime RT-PCR in pak choi sprouts and mature leaves 48 h after application of the respective elicitors. Each value represents the mean ± SD from three biological replicates obtained from semi-quantitative qRT-PCR analysis of gene expression levels after elicitor treatment compared to control plants. Significant changes compared to control are marked with an asterisk (Dunnett test, $p \leq 0.05$).

	**Control**	**MeJA**	**JA**	**LA**	**MeS**
Sprouts					
BrMYB34_1	13.01 ± 0.25	10.25 ± 0.72 *	10.23 ± 0.34 *	12.24 ± 0.97	11.47 ± 0.32 *
BrMYB34_2	11.05 ± 0.42	4.19 ± 0.34 *	4.98 ± 1.21 *	8.02 ± 0.76 *	8.80 ± 0.65 *
BrMYB34_3	6.80 ± 0.18	2.69 ± 0.57 *	3.51 ± 0.30 *	5.98 ± 0.94	5.20 ± 0.25 *
BrMYB51_1	6.81 ± 0.34	6.23 ± 0.93	4.24 ± 1.99 *	5.74 ± 1.51	4.92 ± 0.27
BrMYB51_2	5.31 ± 0.26	4.26 ± 0.49 *	2.50 ± 0.43 *	4.01 ± 0.79 *	3.23 ± 0.27 *
BrMYB51_3	8.56 ± 0.28	5.74 ± 0.05 *	5.01 ± 0.30 *	5.51 ± 1.67 *	6.23 ± 0.67 *
BrMYB51_4	10.30 ± 0.23	7.97 ± 0.58 *	7.97 ± 0.39 *	7.76 ± 0.89 *	7.71 ± 0.31 *
BrMYB122_1	7.78 ± 0.36	4.71 ± 0.12 *	3.73 ± 0.78 *	5.72 ± 1.07 *	5.97 ± 0.51 *
BrMYB122_2	7.10 ± 0.29	5.69 ± 0.23 *	5.47 ± 0.29 *	5.96 ± 1.30 *	5.72 ± 0.27 *
BrCYP79B2_1	8.54 ± 0.46	4.20 ± 0.65 *	4.69 ± 0.43 *	5.95 ± 0.92 *	6.68 ± 0.30 *
BrCYP79B2_2	8.72 ± 0.31	5.43 ± 1.13 *	4.88 ± 0.38 *	7.18 ± 0.88 *	6.91 ± 0.34 *
BrCYP79B3	7.23 ± 0.43	4.23 ± 0.89 *	4.72 ± 0.34 *	5.50 ± 1.28	5.80 ± 0.45
BrSOT16	8.24 ± 0.42	4.69 ± 0.60 *	4.19 ± 0.63 *	6.20 ± 0.52 *	6.20 ± 0.38 *
BrCYP81F1	9.24 ± 0.63	7.42 ± 0.18 *	4.62 ± 0.24 *	6.17 ± 1.41 *	7.30 ± 0.96 *
BrCYP81F2	10.55 ± 0.36	7.68 ± 0.42 *	6.69 ± 0.48 *	7.97 ± 1.78	8.67 ± 0.55
BrCYP81F3_1	6.28 ± 0.23	3.74 ± 0.42 *	4.00 ± 0.36 *	4.98 ± 1.14 *	5.25 ± 0.72
BrCYP81F3_2	6.53 ± 0.20	5.69 ± 0.20	4.75 ± 0.16 *	6.24 ± 1.52	6.51 ± 0.75
BrCYP81F4_1	7.53 ± 0.61	2.48 ± 0.65 *	1.99 ± 0.37 *	5.49 ± 1.63 *	5.99 ± 0.84
BrCYP81F4_2	8.52 ± 0.40	0.72 ± 0.75 *	1.48 ± 0.44 *	5.50 ± 1.38 *	6.01 ± 0.63 *
BrOMT_1	8.31 ± 0.51	2.95 ± 0.99 *	3.25 ± 0.44 *	5.51 ± 1.77 *	6.27 ± 0.84
BrOMT_2	10.04 ± 0.36	8.72 ± 0.39	7.49 ± 0.60 *	7.47 ± 1.70 *	8.49 ± 0.91
Mature leaves					
BrMYB34_1	14.16 ± 0.89	13.97 ± 0.89	13.15 ± 1.67	14.96 ± 2.80	13.76 ± 1.15
BrMYB34_2	6.29 ± 0.44	6.50 ± 0.68	6.80 ± 3.15	8.75 ± 0.71	8.23 ± 3.26
BrMYB34_3	3.54 ± 0.91	4.00 ± 0.47	-0.62 ± 1.02 *	4.50 ± 0.32	8.48 ± 0.41 *
BrMYB51_1	8.01 ± 1.63	8.73 ± 0.38	7.43 ± 1.11	8.26 ± 0.77	7.51 ± 0.61
BrMYB51_2	3.97 ± 0.35	5.94 ± 0.09 *	6.45 ± 0.41 *	6.52 ± 0.40 *	5.75 ± 0.18 *
BrMYB51_3	11.53 ± 0.75	11.25 ± 0.58	10.76 ± 0.31	10.22 ± 1.19	9.25 ± 0.19 *
BrMYB51_4	10.54 ± 0.39	11.50 ± 0.32 *	11.78 ± 0.27 *	10.47 ± 0.24	9.68 ± 0.44 *
BrMYB122_1	12.01 ± 0.98	12.55 ± 0.66	8.47 ± 1.79 *	13.17 ± 0.33	11.66 ± 1.07
BrMYB122_2	12.38 ± 2.72	9.45 ± 0.51	9.40 ± 0.78	8.96 ± 2.42 *	8.97 ± 0.53 *
BrCYP79B2_1	6.00 ± 0.61	5.89 ± 1.41	3.94 ± 0.16 *	7.44 ± 0.20	6.82 ± 0.31
BrCYP79B2_2	8.95 ± 0.78	8.69 ± 0.99	5.15 ± 0.16 *	9.40 ± 0.54	9.27 ± 0.59
BrCYP79B3	10.52 ± 0.79	9.27 ± 1.19	5.91 ± 0.93 *	8.18 ± 0.87 *	9.25 ± 0.58
BrSOT16	5.45 ± 0.53	2.88 ± 0.14 *	0.37 ± 0.07 *	5.93 ± 0.52	4.69 ± 0.36
BrCYP81F1	14.38 ± 0.35	15.36 ± 0.75	13.23 ± 0.74	15.85 ± 0.78 *	13.68 ± 0.17
BrCYP81F2	14.77 ± 1.83	14.89 ± 1.87	13.76 ± 1.31	17.47 ± 0.07	15.16 ± 2.40
BrCYP81F3_1	6.52 ± 0.61	5.42 ± 0.43 *	5.75 ± 0.31	7.48 ± 0.24 *	5.72 ± 0.39

Table S2. *Cont.*

	Control	MeJA	JA	LA	MeS
BrCYP81F3_2	9.53 ± 0.74	8.98 ± 1.24	9.73 ± 1.02	10.98 ± 1.04	9.75 ± 0.39
BrCYP81F4_1	8.50 ± 0.69	7.03 ± 3.47	7.11 ± 2.86	9.72 ± 0.21	7.66 ± 2.68
BrCYP81F4_2	11.77 ± 0.43	−0.02 ± 0.14 *	0.72 ± 0.53 *	12.55 ± 0.26	10.57 ± 1.12
BrOMT_1	5.00 ± 0.75	0.48 ± 0.49 *	1.00 ± 0.12 *	9.25 ± 2.14 *	6.99 ± 0.44 *
BrOMT_2	14.22 ± 0.73	13.11 ± 0.32	12.48 ± 0.27	15.63 ± 0.55	13.18 ± 1.84

MeJA, methyl jasmonate; JA, jasmonic acid; LA, linolenic acid; MeS, methyl salicylate.

© 2013 by the authors; licensee MDPI, Basel, Switzerland. This article is an open access article distributed under the terms and conditions of the Creative Commons Attribution license (http://creativecommons.org/licenses/by/3.0/).
